# *Cunninghamia lanceolata* Resource Distribution Research, Hotspots and Trends via Bibliometric Analysis

**DOI:** 10.3390/plants15020255

**Published:** 2026-01-14

**Authors:** Huaxue Wu, Jie Huan, Zhoujian He, Liqiong Jiang, Peng Zhu

**Affiliations:** 1 College of Forestry, Sichuan Agricultural University, 211 Huimin Road, Wenjiang District, Chengdu 611130, China; 15680813563@163.com; 2 Yanyuan County Forestry and Grassland Bureau, Liangshan Yi Autonomous Prefecture, Liangshan 615715, China; 3 Enyang District Agriculture and Rural Bureau of Bazhong City, No. 6, 40 Planning Road, Enyang District, Bazhong 636600, China; 15388135911@163.com; 4 College of Life Sciences, Sichuan University, 29 Wangjiang Road, Wuhou District, Chengdu 610064, China; Hezhouj@163.com; 5 Institute of Forestry, Chengdu Academy of Agriculture and Forestry Sciences, 200 Nongke Road, Wenjiang District, Chengdu 611130, China; liqiong_jiang@163.com

**Keywords:** *Cunninghamia lanceolata* (Lamb.) Hook., bibliometric analysis, research hotspots, resource distribution, ecological-economic value

## Abstract

Chinese fir [*Cunninghamia lanceolata* (Lamb.) Hook.] is a fast-growing species widely utilized in construction, industrial raw materials. Owing to its broad application scope, research on Chinese fir is fragmented across multiple disciplines, making it difficult to grasp the overall research context and trends. Following the PRISMA guidelines, we retrieved articles related to Chinese fir published between 1942 and 2024 from Chinese databases (i.e., CNKI, Wanfang Data, and VIP Chinese Journal Database) and the Web of Science Core Collection (WOSCC). After removing duplicate and irrelevant records, a total of 7174 valid records were retained, including 5862 from Chinese databases and 1312 from WOSCC. The PRISMA-screened literature was imported into CiteSpace V.6.2.R4 for bibliometric analysis. Through keyword clustering, burst detection, and timeline mapping, we focused on analyzing the domestic resource distribution, research hotspots, and evolutionary trends of Chinese fir research. The results showed that research publications on Chinese fir have increased year by year, and international research started earlier and is more in-depth, while Chinese research covers a wider scope. Both follow two stages (germination and growth). Chinese research focuses on basic application areas such as seedling cultivation and plantation management; international research emphasizes ecological functions and biomass development. Global research exhibits convergence in the field of eco-environmental interactions; specifically, both domestic and international studies investigate the impacts of climate change (e.g., drought and global warming) and nitrogen deposition on the growth and functional evolution of Chinese fir. This study provides references for researchers, forestry policymakers, and planters.

## 1. Introduction

Chinese fir (*Cunninghamia lanceolata* (Lamb.) Hook.) is a monotypic species belonging to the genus *Cunninghamia* R. Br. of the family Cupressaceae Bartling. As an important fast-growing timber species in southern China, it boasts a cultivation history of over 2000 years [1]. Its distribution range covers 17 provinces and autonomous regions in China south of the Qinling–Huaihe Line, and has expanded to Southeast Asia, South America, Oceania, and North America [2,3,4,5]. As the world’s largest cultivator of Chinese fir, China has established approximately 110,000 km^2^ of Chinese fir plantations, accounting for 12.9% of the country’s total plantation area and supplying as much as 30% of the log resources for China’s timber industry [6,7]. This species exhibits remarkable dual ecological and economic value: its high carbon sequestration capacity makes it a preferred species for carbon sequestration, afforestation and ecological restoration [8], while its wood-characterized by light weight, ease of processing, and corrosion resistance—supports traditional industries such as construction, furniture manufacturing, and papermaking [9]. Recent studies have further uncovered its medicinal potential, with extracts from its branches and leaves demonstrating antibacterial, anti-inflammatory, and antitumor activities [10,11,12]; its derivatives (e.g., essential oils) also find applications in the cosmetics and chemical industries [13,14]. Globally, it has emerged as a sustainable substitute for tropical hardwoods [15], highlighting its international significance.

However, the versatility of Chinese fir has led to the fragmentation of research across multiple disciplines, which hinders a systematic understanding of the hotspots and trends in Chinese fir research. Traditional narrative reviews are limited by selective literature sampling and cannot fully capture the interdisciplinary connections, evolutionary trajectories, and emerging trends within this broad research field [16]. Furthermore, existing studies rarely integrate the actual distribution of Chinese fir resources with quantitative analysis of research trends, resulting in a disconnect between eco-geographical contexts and academic dynamics. These limitations underscore an urgent need for standardized, systematic, and comprehensive analyses that integrate resource characteristics and research trends.

To address this gap, we adopted a dual methodological framework combining bibliometrics with the PRISMA guidelines: bibliometrics enables the visualization of literature structures and dynamics, facilitating the identification of core hotspots, evolutionary pathways, and future directions [17,18], while PRISMA ensures transparency and rigor in the systematic review process [19]. Among mainstream bibliometric tools, three dominate the field: CiteSpace, which integrates keyword clustering, burst detection, and timeline mapping, excels at identifying trends, patterns, and emerging topics, and can intuitively reveal the interactions between research topics and time [20]; VOSviewer demonstrates superior performance in generating high-quality network visualizations from complex datasets but lacks in-depth temporal trend analysis [21]; and the R-based Bibliometrix specializes in mapping research topics and their temporal evolution but has limited depth in cluster interpretation [22]. After a comprehensive comparison, we selected CiteSpace for this study due to its unique capability to integrate topic-time interaction analysis (which addresses the limitations of the other two tools) and its proven effectiveness in forestry [23] and ecology [24]——making it well-suited for analyzing the interdisciplinary and fragmented nature of Chinese fir research.

This study explicitly links two complementary themes: resource distribution mapping and bibliometric trend analysis. The former provides the eco-geographical context (e.g., distribution range, core values) to explain why certain research directions (e.g., nitrogen deposition in China, biomass energy globally) have garnered attention; the latter quantifies global (e.g., climate change, carbon neutrality) and regional (e.g., rural revitalization, timber security) drivers that shape research agendas. Under the guidance of PRISMA, we systematically collected 7174 valid publications from 1942 to 2024, including 5862 from Chinese databases (CNKI, Wanfang Data, and VIP Chinese Journal Database) and 1312 from the Web of Science Core Collection (WOSCC). The specific objectives of this study are as follows: (1) to clarify the spatial distribution characteristics and core values of Chinese fir resources in China; (2) to compare the developmental stages, thematic focuses, and quality disparities between domestic and international research; (3) to identify long-term research hotspots and emerging trends; and (4) to propose targeted future research directions based on the identified knowledge gaps.

Compared with previous bibliometric studies in forestry, the innovative aspect of this study lies in its integrated approach—combining resource ecology with bibliometrics to bridge the disconnect between actual resource data and quantitative analysis of research trends. In this way, it not only fills the gap in systematic reviews of Chinese fir research but also provides a replicable model for interdisciplinary comprehensive research on tree species with dual ecological and economic importance. The findings will serve as an important reference for researchers to avoid redundant work, for forestry policymakers to align strategies with global trends, and for growers to optimize resource utilization—ultimately advancing the sustainable development and ecological conservation of the Chinese fir industry.

## 2. Results

### 2.1. Resource of C. lanceolata

#### 2.1.1. The Main Value of *C. lanceolata*

Chinese fir integrates ecological, economic, and industrial values (Figure S1), which aligns with the core themes identified in the bibliometric analysis. Ecologically, its annual carbon sequestration reaches 41.6–277.4 t/km^2^, exceeding that of most coniferous species [25,26] and thus establishing “Carbon sequestration” as an international research hotspot (Section 2.3.1). The canopy of Chinese fir forests can effectively intercept PM2.5 to improve air quality [27], while the forest-medicine compound management model not only ameliorates forest soil but also balances the economic value of medicinal plants, promoting the coordinated development of ecological protection and rural revitalization [28]. This reflects domestic attention to the practical eco-economic synergies associated with the species.

Industrially, Chinese fir wood and its processing residues can be used to produce high-strength pulp, fiberboard, and biomass fuel [29], which aligns with the international burst trend of research focused on “biomass production” (Section 2.3.2). Chinese fir essential oil (predominantly containing cedrol and thujopsene) and branch-leaf extracts (rich in germacrene D and β-elemene) hold broad application prospects in cosmetics, medicine, and drug development [11,12,13,14]. These findings echo the cluster results related to “volatile compounds” and “extracts” and highlight emerging research directions in medicinal and chemical applications.

Economically and in practical applications, Chinese fir wood is lightweight and easy to process, serving as a high-quality raw material for traditional architecture (e.g., Dong drum towers and flower bridges) and furniture production [30,31,32]. Innovative approaches such as material compounding and structural optimization can further enhance the market acceptance of Chinese fir products [33,34,35,36], which is linked to key domestic research focuses on “sawmilling technology”, “Microfibril Angle (MFA)” and “modulus of elasticity” (Section 2.3.1).

#### 2.1.2. Distribution of *C. lanceolata* in China

Chinese fir is widely distributed across 17 provinces, municipalities directly under the Central Government, and autonomous regions in China south of the Qinling–Huaihe Line, with its geographical range spanning 21.52–34.05° North Latitude (N) and 101.50–121.88° East Longitude (E) [2] (Figure S2). To the north, its distribution extends to the southern foot of the Qinling Mountains, the Tongbai Mountains in Henan, the Dabie Mountains in Anhui, as well as Jurong and Yixing in Jiangsu; to the south, it reaches Xinyi in Guangdong, Yulin and Longjin in Guangxi, and Guangnan, Malipo, Pingbian, Kunming, Huize, and Dali in Yunnan; to the east, it covers southern Jiangsu, Zhejiang, and the western mountainous areas of Fujian; to the west, it extends to the Dadu River Basin in Sichuan (the area east of Moximian in Luding) and the southwestern part of the Anning River Basin [37]. This extensive cultivated distribution also explains why domestic research literature on Chinese fir mostly focuses on “plantations” and “clones” (Section 2.3.1): the context of large-scale cultivation has driven domestic research to focus on localized cultivation techniques, while international research, due to the lack of a research foundation based on the species’ extensive local distribution, places greater emphasis on its global-scale ecological functions (e.g., Carbon sequestration).

#### 2.1.3. Integrated Breeding-Processing Technology System of *C. lanceolata*

The full-life-cycle technical system of Chinese fir encompasses three core stages (Figure S3). The technical key points of each stage are highly consistent with the core keywords identified in the bibliometric analysis, providing systematic support for Chinese fir research and industrial practice.

Seed Breeding and Seedling Raising focuses on germplasm quality improvement. Superior mother trees aged 15–30 years with robust growth are selected, and seeds are collected when the cones ripen in October–November. After being disinfected with potassium permanganate solution, the seeds are stored under low-temperature conditions [38]. Nursery sites are preferably located in mixed forest areas with fertile soil and convenient drainage. Following germination promotion treatment, the seeds are subjected to low-temperature stratification at 5 °C to improve the germination rate [39,40,41]. Seedling raising can be conducted via drill seeding or light substrate mesh bag seedling raising technology [42,43]. The technical focus of this stage is precisely consistent with the domestic cluster keywords of “clones” and “sprout tillers”, highlighting the core domestic research direction of Chinese fir germplasm optimization.

Field Management adopts a stand age-specific refined management strategy. Young forests are mainly managed through intertillage and weeding, supplemented by soil loosening and pit expansion. Thinning of mature forests strictly follows the principles of “retaining large, sparse, superior, and straight trees” while simultaneously removing diseased, weak, and dead plants [44,45]. Water and fertilizer management requires scientific regulation of fertilizer dosage, type, and application time to avoid root burn [46]. Pest and disease control relies on integrated measures combining biological and chemical control methods [47]. The technical points of this stage are highly consistent with research hotspots such as “nitrogen deposition” and “soil”, as precision nutrient management is key to alleviating environmental stress and maintaining stand health.

Harvesting and processing specify a 20–25-year rotation period for Chinese fir, and logged timber is graded according to diameter after felling [48]. The processing flow includes, in sequence, debarking (to remove bark and impurities) [49], grading (classified by Young’s modulus/moisture content to optimize lumber yield) [50], drying (to improve wood dimensional stability) [51], and modification (to expand application scenarios) [52]. For small-diameter wood and thinned wood, resource utilization efficiency can be improved through modification and composite technologies [53]. The technology in this stage is associated with cluster keywords such as “sawmilling technology”, “modulus of elasticity”, and “Microfibril Angle (MFA)”, effectively addressing the pain point of low-value utilization of small-diameter wood and thinned wood.

### 2.2. Analysis of the Overall Situation of Literature

#### 2.2.1. Annual Distribution Characteristics of the Number of Publications

**Figure 1:**
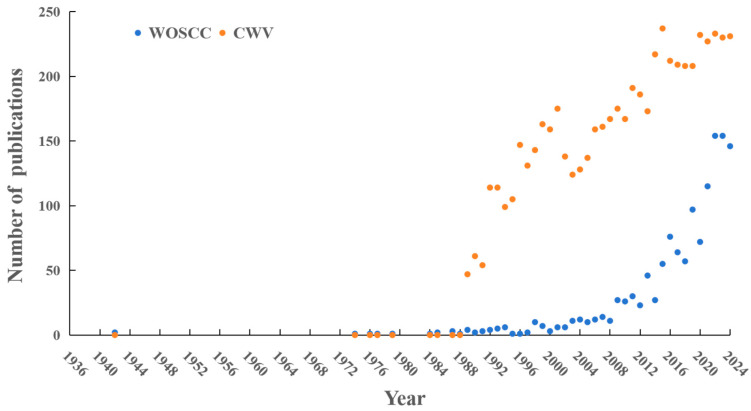
Annual trends in the number of *C. lanceolata* publications. CWV: CNKl (https://www.cnki.net/, accessed on 2 January 2025), Wanfan data (https://w.wanfangdata.com.cn/index.html?index=true, accessed on 2 January 2025), and VIP data (http://www.cqvip.com/, accessed on 2 January 2025) about publications of *C. lanceolata*. WOSCC: Web of Science core collection about publications of *C. lanceolata*.

The temporal distribution of the number of published papers can reflect the development speed and phased changes in a research field to a certain extent, and is of great significance for predicting development trends and judging research dynamics [54]. Based on Figure 1, a systematic analysis of Chinese fir-related literature in China’s three major databases and the WOSCC database revealed that the WOSCC database first included Chinese fir research literature in 1942, 47 years earlier than China’s domestic databases (1989), highlighting the early attention paid by the international academic community to this China-endemic tree species. Its publication trend can be divided into two distinct stages: the germination stage (1942–2004), characterized by an average of fewer than 5 papers published per year and unstable publication volume, indicating that Chinese fir had not yet become a mainstream research focus during this period; and the growth stage (2008–2024), during which the number of publications increased at an average annual rate of 9.2%, reaching a peak of 154 papers in 2022. Although the total number of English-language publications on Chinese fir is generally less than that of Chinese-language publications, it shows an overall upward trend, reflecting the accelerated improvement in the internationalization level of research in this field. Domestic research on Chinese fir began in 1989, and its research progress can also be divided into two stages. During the initial development stage (1989–2008), the number of published papers increased gradually, showing an overall upward trend despite fluctuations, which indicated that domestic attention to Chinese fir research began to grow, the number of researchers gradually increased, and the research scope continued to expand. In the stage of rapid growth and stability (2009–2024), the number of publications increased significantly, reaching a historical peak of 257 papers in 2015 and stabilizing at around 230 papers after 2020. This reflects the maturing of the domestic Chinese fir research system, with a large volume of achievements being produced and stably maintained at a high level. These trends highlight the vigorous development momentum and broad exploration potential of research in this field on a global scale.

#### 2.2.2. Journal Rank

The rank of research journals is a key indicator for measuring research quality [55]. Table 1 presents in detail the information on the top 10 journals with the highest publication volume on Chinese fir research at home and abroad, and reveals their differentiated characteristics by integrating impact factors, subject classifications, and JCR quartile attributes. In the international journal (WOSCC) segment, JCR Q1 quartile journals occupy a core dominant position, covering multidisciplinary journals such as Forest Ecology and Management, Plant and Soil, and Science of the Total Environment. Their subject scope extends to plant science, environmental science, and other fields, with generally high impact factors (e.g., Science of the Total Environment reaches 8.0), fully reflecting the interdisciplinary nature and strong academic influence of international Chinese fir research.

Domestic journals (CWV), by contrast, are highly focused on the field of forestry. Among them, Scientia Silvae Sinicae and Forest Research rank among the top with publication shares of 17.8% and 13.4%, respectively. These journals place greater emphasis on applied research on domestic Chinese fir resources; however, constrained by factors such as the scope of international dissemination and the diversity of manuscript sources, the impact factors of most domestic journals are relatively low (e.g., Journal of Fujian Forestry Science and Technology is only 0.890), indicating that domestic journals in the field of Chinese fir research still have significant room for improvement in terms of international academic discourse power.

Overall, the differences between domestic and international journals not only reflect the divergence in research focus—international journals lean toward interdisciplinary frontier exploration, while domestic journals concentrate on localized applied research—but also mirror the distinct developmental stages of the two in terms of academic communication and influence.

### 2.3. Research Trend and Hotspot Analysis

#### 2.3.1. Keyword Clustering Analysis and Temporal Distribution

**Figure 2:**
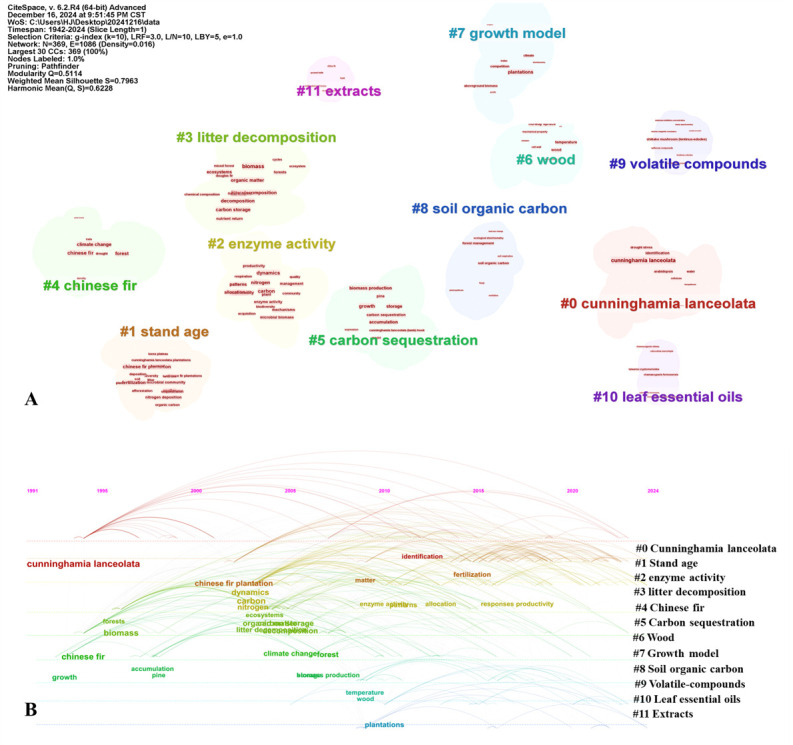
Keyword timing distribution and cluster analysis of *C. lanceolata* in the WOSCC database. (**A**) Keyword clustering of *C. lanceolata*. (Q = 0.5114), Silhouette (S = 0.7963) (**B**) Timeline chart of key words of *C. lanceolata.*

Keywords are intuitive and concise representations of the core themes of academic papers. High-frequency keyword cluster analysis can reveal stable research hotspots, while burst analysis identifies rapidly emerging research trends; the combination of the two comprehensively presents the research hotspots and future development trends of the field [56,57]. To clarify the global and domestic patterns of Chinese fir research, this study systematically analyzed the high-frequency keywords in the WOSCC database (international literature) and China’s three major databases (domestic literature). The results are as follows:

A total of 12 valid clusters were generated from the WOSCC database (Figure 2A), which can be categorized into four types based on research content: the first category is the research on Chinese fir resources, including cluster #0, “*Cunninghamia lanceolata*” and cluster #4, “Chinese fir”. The timeline of these clusters is very extensive (Figure 2B), indicating that resource-related issues have become an important foundation for plant research. The second category is the research on the ecological functions of Chinese fir, which mainly includes: cluster #1, “stand age”, cluster #3, “litter decomposition”, cluster #5, “Carbon sequestration”, cluster #7, “growth model” and cluster #8, “soil organic carbon”. The third category is the research on the physiological mechanisms of Chinese fir, consisting of cluster #2, “enzyme activity”. The fourth category is the research on the development and utilization of Chinese fir, composed of the remaining clustering modules. As a high-frequency word, cluster #6, “wood” highlights the industrial value of Chinese fir as an economic tree species, and the related research between cluster #9, “volatile compounds” and cluster #11, “extracts” implies its potential applications in the fields of biomedicine and green materials. Overall, international Chinese fir research is dominated by ecological function studies and is gradually expanding toward the utilization of secondary metabolites.

The keyword clustering of the three major domestic databases also yielded 12 valid clusters (Figures S4 and S5), which can be divided into 3 research directions, and the results are significantly different from those of international literature (Figure 2). The first category is the research on resource cultivation of Chinese fir, including cluster #0, “*Cunninghamia lanceolata*”, cluster #1, “plantation”, cluster #5, “clone” and cluster #8, “sprout tillers”, covering basic research on species, plantation cultivation and clonal propagation. The second category is the research on the ecological environment of Chinese fir, mainly including: cluster #2, “nitrogen deposition”, cluster #3, “*C. lanceolata* forest”, cluster #4, “biomass” and cluster #7, “soil”, exploring regional environmental issues such as nitrogen deposition in southern China. The third category is the research on wood utilization of Chinese fir, consisting of cluster #9, “modulus of elasticity”, cluster #10, “sawmilling technique” and cluster #11, “Microfibril Angle (MFA)”, which deeply explores wood mechanical properties, optimization of processing technology and characteristics of microstructure, showing an overall situation where basic research and application practice are equally emphasized. A unique feature of domestic clustering is Cluster #6 “*Pinus massoniana* Lamb”, which is often studied in comparison with Chinese fir. As the main afforestation tree species in southern China, their ecological niches and distribution areas overlap [58]. Comparative studies can optimize the configuration of mixed forests, guide the differentiated utilization of wood, and provide a supplementary perspective for Chinese fir research. Overall, domestic research on Chinese fir focuses on resource cultivation, ecological environment, and wood utilization, among which ecological environment research is a key domestic hotspot. Both international and Chinese research on the Chinese fir focus on the field of ecological functions and environmental interaction mechanisms.

#### 2.3.2. Keyword Burst Analysis

**Figure 3:**
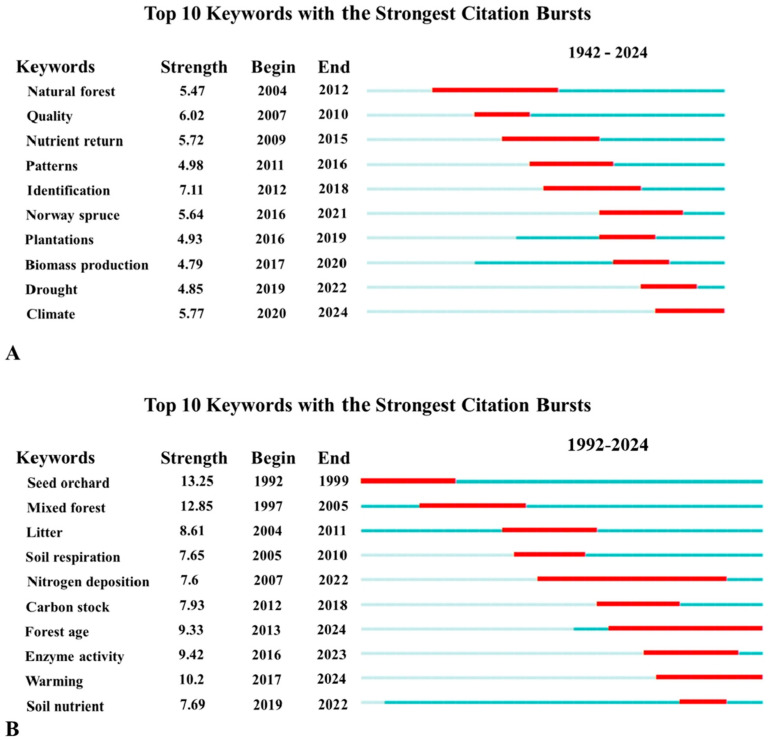
Keywords with the strongest citation bursts of *C. lanceolata*. (**A**) Keywords from 1942 to 2024 appear in WOSCC. (**B**) The keywords of 1992–2024 in the three major Chinese databases appear. The red portion indicated that the keyword was in high frequency during the time period.

Keyword bursts can identify burst terms in different time periods, thereby analyzing research hotspots in different time periods and predicting future research directions [59]. This study conducted a comprehensive analysis of the strongest citation bursts of keywords in domestic and international databases, with the results shown in Figure 3.

In the clustering results of English literature (Figure 3A), the phased characteristics of international Chinese fir research are precisely consistent with the literature publication trend: No significant burst terms were detected before 2004, which aligns with the germination period of literature (1942–2004)—an average of fewer than 5 papers were published annually with unstable quantities, indicating that Chinese fir had not yet become a mainstream research object and confirming the field was in an exploratory stage without concentrated hotspots. After 2005, the literature entered a growth period (average annual growth rate of 9.2%), and the first batch of research hotspots emerged simultaneously. Among them, “Quality (wood quality)” had a burst intensity of 6.02, higher than 5.47 of “natural forests”, indicating that improving wood quality was the core focus at that time. In the past five years, “Identification” has become the hottest theme with a high burst intensity of 7.11, accompanied by the appearance of “Norway spruce (Picea abies)”, reflecting that international research has expanded to a comparative perspective between Chinese fir and congeneric tree species. Although “Climate” did not have the highest burst intensity, it was the only keyword continuously bursting until 2024, marking that international Chinese fir research has shifted from scattered themes and single dimensions to comprehensive application directions focusing on climate response.

The burst term results of Chinese literature (Figure 3B) are deeply matched with the development process of domestic literature, further highlighting the depth and pertinence of localized research. During the initial development stage (1989–2008), the number of studies gradually increased, and research attention continued to rise. In this stage, “Seed orchard” reached the highest burst intensity of 13.25 in the entire figure, intuitively reflecting the high attention paid to the foundation of Chinese fir germplasm in early domestic research, which exactly matched the urgent demand for improved varieties in forestry production at that time. After 2009, the research entered a stage of rapid growth and stability, with the literature system becoming mature and achievements stably maintained at a high level. The 15-year burst cycle of “Nitrogen deposition” is a direct manifestation of the long-term and systematic in-depth research conducted by the domestic academic community on the correlation between nitrogen input and Chinese fir forest ecosystems during this period. In recent years, against the background of the number of studies stably maintained at around 230, “Warming” has a burst intensity of 10.2. Combined with the continuous activity of “Forest age” until 2024, it not only reflects that domestic research keeps up with global climate hotspots but also deeply integrates climate change response with the inherent structural characteristics of Chinese fir forests (i.e., forest age), making the research more targeted. Thus, while aligning with international trends, domestic Chinese fir research has formed a differentiated in-depth research field.

## 3. Discussion

### 3.1. Intrinsic Correlation Between Chinese Fir Resource Distribution and Multidimensional Values

The extensive distribution of Chinese fir across 17 provinces/municipalities/autonomous regions in southern China (21.52–34.05° N, 101.50–121.88° E) is essentially the synergistic outcome of its strong ecological adaptability and prominent economic and social values. Ecologically, it exhibits broad tolerance to subtropical to warm temperate climates (annual precipitation: 800–2000 mm; annual average temperature: 15–23 °C), acidic red/yellow soils with a pH of 4.5–6.5, and mountainous/hilly terrain, coupled with high tolerance to poor soil conditions—laying a solid biological foundation for its trans-regional distribution [60,61]. Economically, as the most important fast-growing timber species in southern China, its wood supplies 30% of the national industrial logs, serving as a strategic resource for safeguarding national timber security. Its processed products cover traditional industries such as construction, furniture, and papermaking, forming a complete industrial chain [7,62]. Ecologically, Chinese fir forests achieve an annual carbon sequestration capacity of 41.6–277.4 t/km^2^, significantly higher than other coniferous species [20,21]. As a core component of the ecological barriers in the Yangtze and Pearl River basins, they play a crucial role in soil and water conservation, climate regulation, and air purification [63]. This virtuous cycle of “wide distribution—high value—strong demand” not only explains the fundamental reason for its long-term status as a research hotspot but also provides an eco-economic synergistic theoretical basis for the optimal allocation of resources in subsequent studies.

### 3.2. Differences in Research Hotspots (China vs. International) and Cause Analysis

Based on a comprehensive bibliometric analysis of Chinese and international literature, research on Chinese fir presents a pattern of “global consensus coexisting with regional characteristics”: the core consensus focuses on ecological functions and environmental interaction mechanisms, yet there are significant differences in research focuses between Chinese and international studies. The causes are closely related to the resource conditions of various countries, regional needs, and common global challenges.

Chinese research centers on three core themes: resource cultivation, ecological response, and wood utilization (keyword clusters: plantation, nitrogen deposition, sawmilling technique), which is highly consistent with China’s national context as the main planting area of Chinese fir [5]. China’s total nitrogen deposition is 3–5 times that of European countries and far exceeds the natural nitrogen load [64], while the distribution area of Chinese fir happens to be mostly regions with high nitrogen deposition rates [65]. Therefore, the response of Chinese fir plantation ecosystems to nitrogen input has attracted considerable attention from the academic community [66,67,68]. Existing studies have shown that nitrogen addition can improve soil nitrogen availability and alter the nutrient balance (C/P and N/P ratios) of Chinese fir plantations, which may further affect soil microbial community composition [69,70] and plant nutrient acquisition strategies [71]. Due to the multiple significant impacts of nitrogen deposition on Chinese fir ecosystems, “nitrogen deposition” has remained a research hotspot in China for 15 consecutive years. Relevant studies have systematically revealed the mechanisms by which nitrogen input affects Chinese fir seedling growth, soil microbial communities, and ecosystem stability [72]. In addition, China boasts a large-scale Chinese fir plantation (approximately 110,000 square kilometers) but has long faced the practical problem of low utilization rate of small-diameter wood [6,53]. This has promoted the in-depth development of application-oriented research, such as wood modification and processing technology optimization, and has also shaped the distinct “problem-oriented” characteristic of Chinese fir research in China.

International research focuses on three core themes: quantification of ecological functions, response to global changes, and development of biomass energy (keyword clusters: carbon sequestration, climate, biomass production). On the one hand, lacking the extensive local planting background of China, international studies pay more attention to the universal value of Chinese fir in global ecosystems, such as its key role as a carbon sink in subtropical forests [73]. On the other hand, the global energy structure transformation and climate crisis have driven the international academic community to focus on the biomass energy potential of Chinese fir [74], as well as the impacts of extreme climates like drought and warming on its geographical distribution and growth characteristics [75], forming a trend-oriented research feature. This difference is not contradictory but complementary. Chinese research provides localized empirical data support for the international academic community, while international research points out the global research and development direction for China. The two join forces collaboratively to promote Chinese fir research toward deeper levels and broader fields.

### 3.3. Limitations of Bibliometric Research

Although this study’s bibliometric analysis based on Chinese and English databases can reveal the overall trends in Chinese fir research, it still has certain limitations that should be viewed objectively. On the one hand, there is a language bias, as only literature from Chinese databases (CNKI, Wanfang, VIP) and English databases (WOSCC) was included. Chinese fir is also cultivated and studied in non-Chinese and non-English speaking countries such as Brazil, Vietnam, and New Zealand [3,4,5], and the exclusion of literature from these regions may lead to the omission of some research hotspots. On the other hand, the database coverage is incomplete. It neither includes document types such as government reports, technical specifications, and unpublished dissertations, nor retrieves specialized databases like the Vietnamese Agricultural Science Journal Database and the Brazilian Forestry Database, which may affect the comprehensiveness of the research. In addition, there are limitations in keyword extraction. CiteSpace identifies hotspots based on keyword co-occurrence and burst detection, but the core content of some studies may not be reflected in keywords. Differences in the expression of the same concept among different researchers may also reduce the accuracy of clustering results. These limitations provide directions for improvement in future bibliometric research, namely expanding the coverage of languages and databases, and integrating subject terms with abstract semantic analysis to further enhance the comprehensiveness and accuracy of research results.

### 3.4. Implications

Based on a comparative analysis of Chinese and international literature, this study identifies two key knowledge gaps in Chinese fir research: the insufficient exploration of ecological adaptation mechanisms at the molecular level, and the lack of synergy and cross-regional collaborative research between Chinese and international studies. This conclusion points out future directions for researchers. Building on existing research in resource cultivation, ecological response, and wood utilization, domestic researchers can further deepen the study of the interaction mechanisms between Chinese fir and the local environment, such as investigating the molecular mechanisms of Chinese fir’s response to nitrogen deposition and screening functional genes related to nitrogen use efficiency. Meanwhile, drawing on international research methods and ideas, they should conduct studies on global issues such as Chinese fir’s adaptation to climate change and biomass energy development to enhance the international influence of domestic research. International researchers, on the other hand, can strengthen cooperation with Chinese researchers, leveraging China’s large-scale planting background and abundant empirical data to conduct in-depth research on the universal laws of Chinese fir, thereby promoting the advancement of global Chinese fir research.

Forestry managers and policymakers can obtain practical guidance from the research findings to support the sustainable development of the Chinese fir industry. Forestry managers should formulate targeted management strategies based on the ecological adaptability and environmental response rules of Chinese fir: for instance, improving the soil nitrogen-phosphorus ratio by reducing nitrogen fertilizer application and increasing phosphorus and potassium fertilizer inputs to optimize the growth environment of Chinese fir and improve its yield [76]; in terms of planting layout, adjusting the planting area and variety selection according to the ecological adaptability of Chinese fir and climate change prediction results to enhance the stability and sustainability of Chinese fir forests. For example, in areas facing drought risks due to climate change, drought-tolerant Chinese fir varieties should be selected for planting. In addition, strengthen the management of small-diameter wood, promote the application of wood modification and processing technology optimization, and improve the utilization rate and economic value of small-diameter wood [53].

Policymakers should formulate targeted policies to promote the sustainable development of the Chinese fir industry by integrating its dual ecological and economic values: at the ecological protection level, encourage the development of Chinese fir carbon sink forests and promote the integration of their carbon sink functions into the forestry carbon trading system to facilitate the achievement of carbon peaking and carbon neutrality goals [77]; meanwhile, formulate nitrogen emission control policies to mitigate the negative impacts of nitrogen deposition on Chinese fir ecosystems. At the industrial development level, support the research, development, and promotion of wood modification and processing technologies, and encourage the high-value utilization of Chinese fir wood to drive industrial upgrading. Furthermore, combined with the rural revitalization strategy, develop forest tourism and health and wellness industries relying on the landscape value of Chinese fir forests [78], support the development of the under-forest economy, and open up new channels for farmers’ income increase, ultimately realizing the coordinated advancement of ecological protection and economic development [79].

## 4. Materials and Methods

### 4.1. Research Design

This systematic review adhered to the PRISMA 2020 guidelines [77] to ensure transparency and rigor. We integrated literature retrieval from Chinese and international databases, followed by data deduplication with EndNote X9 (Clarivate Analytics, Philadelphia, PA, USA) and bibliometric analysis with CiteSpace V.6.2.R4 (Drexel University, Philadelphia, PA, USA), aiming to systematically explore Chinese fir research trends and resource distribution.

### 4.2. Data Collection

#### 4.2.1. Databases and Retrieval Strategy

Literature retrieval was conducted on 2 January 2025, covering two types of databases:

International database: Web of Science Core Collection (WOSCC), with the search keyword “*Cunninghamia lanceolata*”, restricted to English language and journal article document type.

Chinese databases (CWV): China National Knowledge Infrastructure (CNKI https://www.cnki.net/, accessed on 2 January 2025), Wanfang Database (https://w.wanfangdata.com.cn/index.html?index=true, accessed on 2 January 2025), and Weipu Database (VIP http://www.cqvip.com/, accessed on 2 January 2025), with the search term “shamu” in the subject or title, restricted to Peking University Core Journals.

#### 4.2.2. Inclusion and Exclusion Criteria

Inclusion: All publications related to *Cunninghamia lanceolata*, published between 1942 and 2024, with complete abstracts or full texts.

Exclusion: Duplicate records; non-journal publications (e.g., conference papers, theses, book chapters); irrelevant studies (e.g., mentions of Chinese fir only as a secondary reference).

#### 4.2.3. Data Cleaning

Duplicates were removed using EndNote X9: first by automatic deduplication based on title, authors, and source journal, then manual verification to eliminate false duplicates across the three Chinese databases. After screening, 7174 valid records were retained (5862 from Chinese databases, 1312 from WOSCC).

### 4.3. Data Analysis

#### 4.3.1. PRISMA Flowchart Explanation

**Figure 4:**
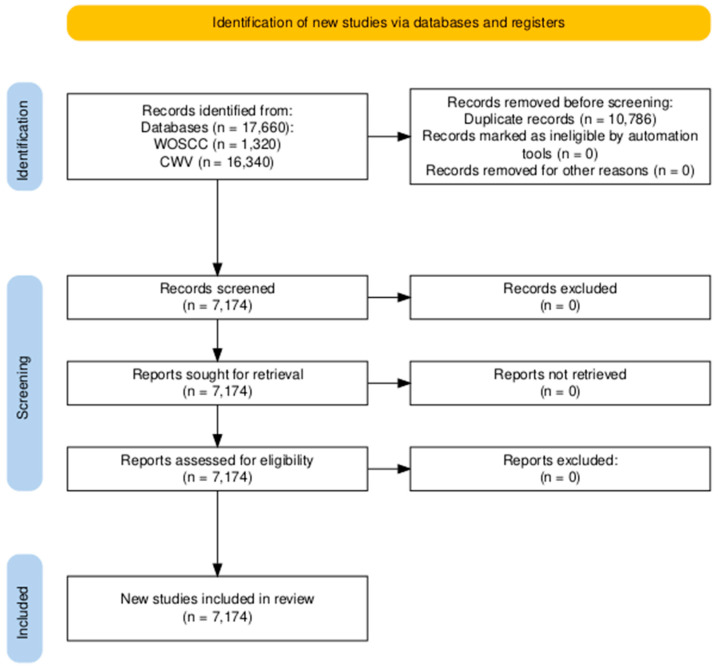
The process of literature analysis. Source: [19]. CWV: CNKI (https://www.cnki.net/, accessed on 2 January 2025), Wanfang data (https://w.wanfangdata.com.cn/index.html?index=true, accessed on 2 January 2025), and VIP data (http://www.cqvip.com/, accessed on 2 January 2025) about publications of *C. lanceolata*. WOSCC: Web of Science core collection about publications of *C. lanceolata*.

Figure 4 outlines the full literature processing workflow: 17,660 initial records were retrieved (16,348 from Chinese databases, 1312 from WOSCC; 10,486 duplicate and irrelevant records were excluded, leaving 7174 records for eligibility assessment; no additional records were excluded after full-text review, as all met the predefined inclusion criteria; and finally, 7174 valid records were included in the final bibliometric analysis.

#### 4.3.2. Bibliometric Analysis with CiteSpace

CiteSpace V.6.2.R4 was used for visualization, with parameter settings based on standard forestry bibliometric practices [20,23,55]: time slicing was set to the period 1942–2024 with a 1-year slice width; the g-index was configured as k = 25, a value that balances network density and clarity for large datasets; network pruning was implemented using the Pathfinder and slicing network pruning algorithms to enhance cluster distinctiveness; keyword extraction was performed via the Log-Likelihood Ratio (LLR) algorithm, with clustering results required to meet the thresholds of modularity > 0.3 and silhouette > 0.5 (an indicator of reliable clustering); and the analysis covered four core dimensions, namely annual publication trends, journal rank, keyword clustering, and burst detection.

## 5. Conclusions

This study integrated bibliometric methods with resource ecology to systematically analyze 7174 valid publications on Chinese fir from 1942 to 2024, revealing the species’ resource distribution characteristics, global research hotspots, and evolutionary trends. The main findings are summarized as follows: (1) Chinese fir is widely distributed across 17 regions in southern China, boasting strong ecological adaptability and dual ecological-economic values, forming a synergistic “distribution-resource-value” effect. (2) International research began earlier (in 1942) and focuses primarily on ecological functions (e.g., carbon sequestration, climate response) and biomass energy development; domestic research (starting in 1989) covers resource cultivation, regional ecological responses (e.g., nitrogen deposition), and wood utilization, presenting a pattern of “global consensus + regional characteristics”. (3) Driven by global climate change, national strategic demands, and industrial practical needs, both Chinese and international research hotspots converge on the interaction mechanisms between ecological functions and the environment.

Despite its contributions, this study has limitations, including language bias, incomplete database coverage, and constraints on keyword extraction——these also clarify directions for future methodological optimization. Based on the identified knowledge gaps and research trends, targeted future research directions are proposed: first, to address the insufficient exploration of ecological adaptation mechanisms at the molecular level, employ modern molecular biology techniques such as genomics and transcriptomics to deeply analyze the molecular mechanisms of Chinese fir’s response to key environmental factors, and identify core genes regulating its ecological adaptability; second, to tackle the lack of synergy between Chinese and international research, promote cross-regional collaborative studies with Chinese fir-cultivating countries such as Brazil and Vietnam, establish a Sino-foreign scientific research cooperation platform, integrate empirical data from China’s large-scale plantations with international cutting-edge research methods, and conduct global-scale collaborative research on Chinese fir’s resource distribution, ecological functions, and environmental interactions, advancing the field toward deeper and broader dimensions. In summary, this study provides a scientific reference for Chinese fir-related research, management, and policy formulation, supporting the sustainable development of the Chinese fir industry and the construction of ecological civilization.

## Figures and Tables

**Table 1:** Top 10 Journals by publication count for *C. lanceolata* research.

Type	Journal	Impact Factor	Categories	Number
WOSCC	Forests	2.5	Forestry	36.9%
	Forest ecology and management	3.7	Forestry	15.7%
	Plant and soil	4.1	Agronomy, plant sciences, and soil science	8.8%
	Journal of Forestry Research	4.6	Forestry	6%
	Bioresources	1.6	Materials science, paper & wood	5.8%
	Frontiers in Plant Science	4.8	Plant sciences	5.8%
	Plos One	2.6	Multidisciplinary sciences	5.8%
	Science of the total environment	8	Environmental sciences	5.6%
	Scientific reports	3.9	Multidisciplinary sciences	4.7%
	Trees structure and function	2.1	Forestry	4.7%
CWV	Scientia silvae sinicae	2.692	Forestry	17.8%
	Forest research	3.072	Forestry	13.4%
	Journal of forest and environment	3.317	Forestry	12.2%
	Acta ecologica sinica	7.268	Biology	10.9%
	Journal of central south university of forestry & technology	4.033	Forestry	10.5%
	Journal of Fujian forestry science and technology	0.890	Forestry	8.2%
	Journal of Zhejiang forestry science and technology	1.129	Forestry	8%
	Journal of Northeast Forestry University	2.216	Forestry	6.8%
	Chinese journal of applied ecology	5.682	Agribusiness	6.4%
	Journal of Beijing Forestry University	3.058	Forestry	6%

Note: The impact factors and subject categories of international journals were obtained through the WOSCC. The impact factors listed were as of the year 2024, and where multiple subject categories are available, the one with the highest ranking in the Journal Citation Reports (JCR) was selected. Journals highlighted in red were those classified in the Q1 zone of the JCR. The acronym “CWV” represents a composite of the China National Knowledge Infrastructure (CNKI) (https://www.cnki.net/, accessed on 2 January 2025), Wanfang (https://w.wanfangdata.com.cn/index.html?index=true, accessed on 2 January 2025), and VIP (http://www.cqvip.com/, accessed on 2 January 2025). The impact factors and subject categories for journals were retrieved via the CNKI platform.

## Data Availability

The original contributions presented in this study are included in the article/Supplementary Materials. Further inquiries can be directed to the corresponding author.

## Supplementary Materials

The following supporting information can be downloaded at https://www.mdpi.com/article/10.3390/plants15020255/s1. Figure S1: The main value of *C. lanceolata*; Figure S2: Distribution of *C. lanceolata* in China; Figure S3: Flow chart of *C. lanceolata* breeding to processing; Figure S4: Keyword clustering of *C. lanceolata* in the three major Chinese databases; Figure S5: Keyword timing distribution of *C. lanceolata* in the three major Chinese database.
