# Supplementary material for: Cunninghamia lanceolata Resource Distribution Research, Hotspots and Trends via Bibliometric Analysis
Source: Plants (Basel). 2026 Jan 14;15(2):255. doi: 10.3390/plants15020255 (PMC12845276; doi:10.3390/plants15020255)
Supplement: Supplementary file 1 [file plants-15-00255-s001.zip › plants-4075525-supplementary.pdf]

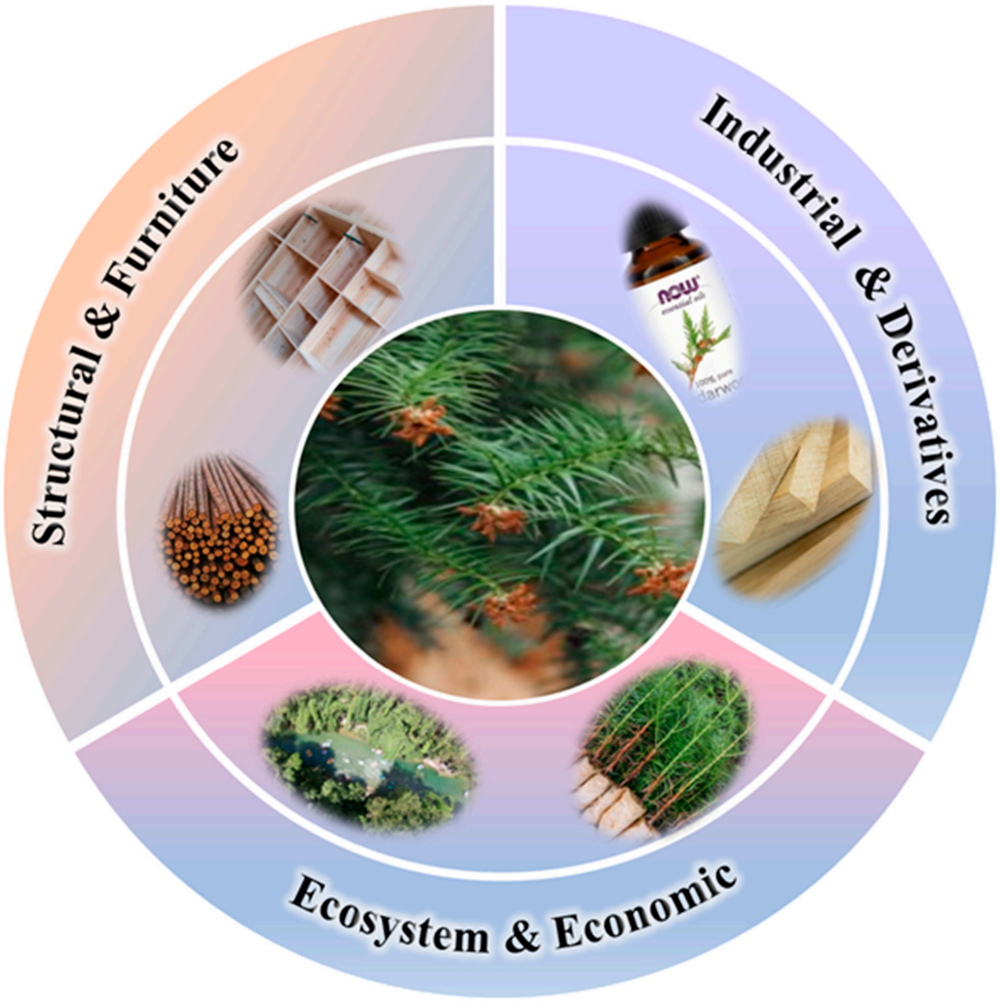

Figure S1 The main value of *C. lanceolata*.

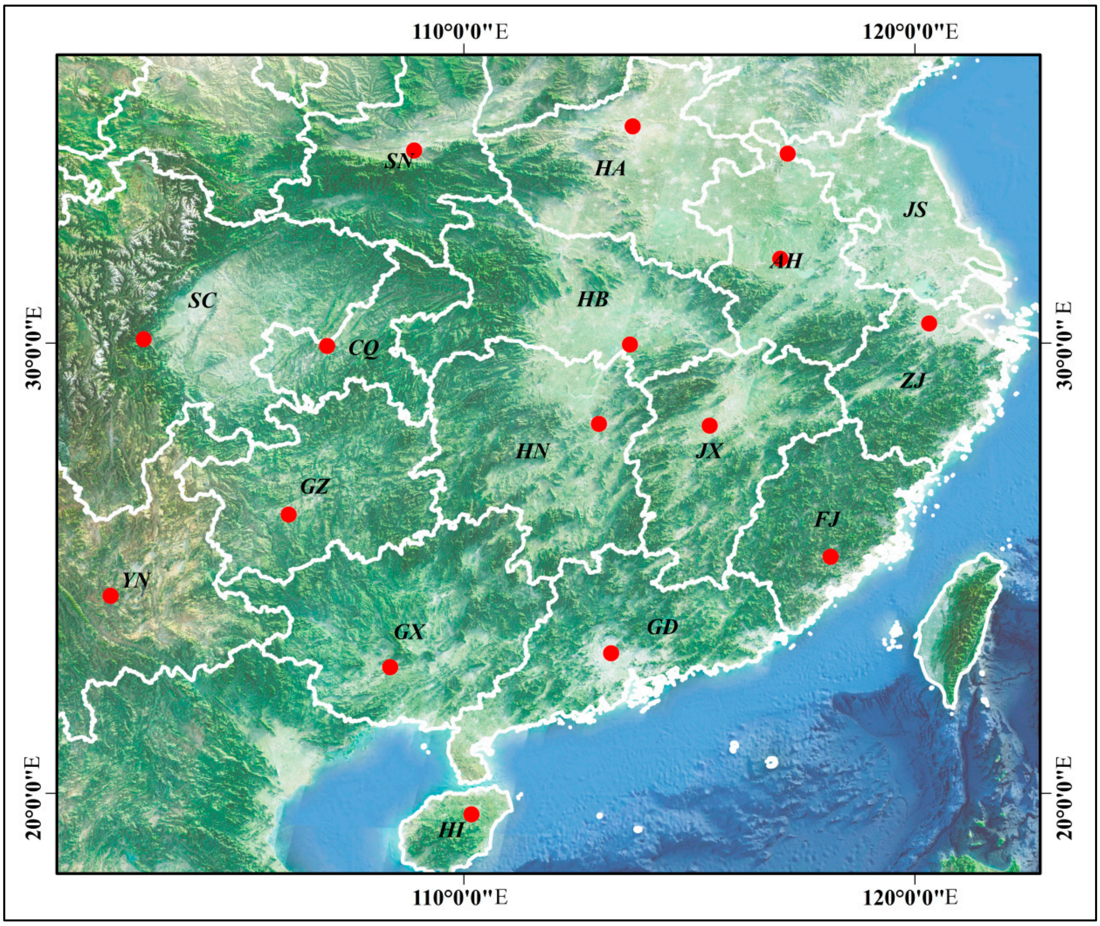

Figure S2 Distribution of *C. lanceolata* in China. The red dots in the figure indicate the distribution points of *C. lanceolata* across various provinces. SC: Sichuan province; YN: Yunnan province; SN: Shaanxi province; CQ: Chongqing province; GZ: Guizhou province; HA: Henan province; HB: Hubei province; HN: Hunan province; GX: Guangxi Zhuang autonomous region; AH: Anhui province; JX: Jiangxi province; GD: Guangdong province; JS: Jiangsu province; ZJ: Zhejiang province; FJ: Fujian province; HI: Hainan province. The image was created using ArcGIS 10.5.

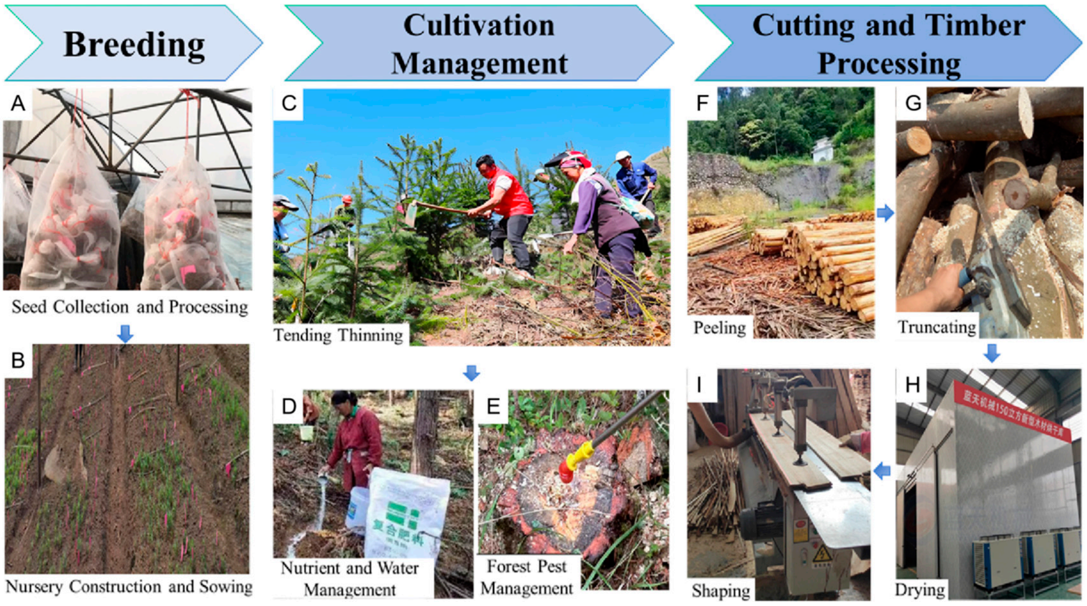

Figure S3 Flow chart of *C. lanceolata* breeding to processing. All visual images are obtained from publicly available online sources. Achieved from https://image.baidu.com, accessed on 22 March 2025.

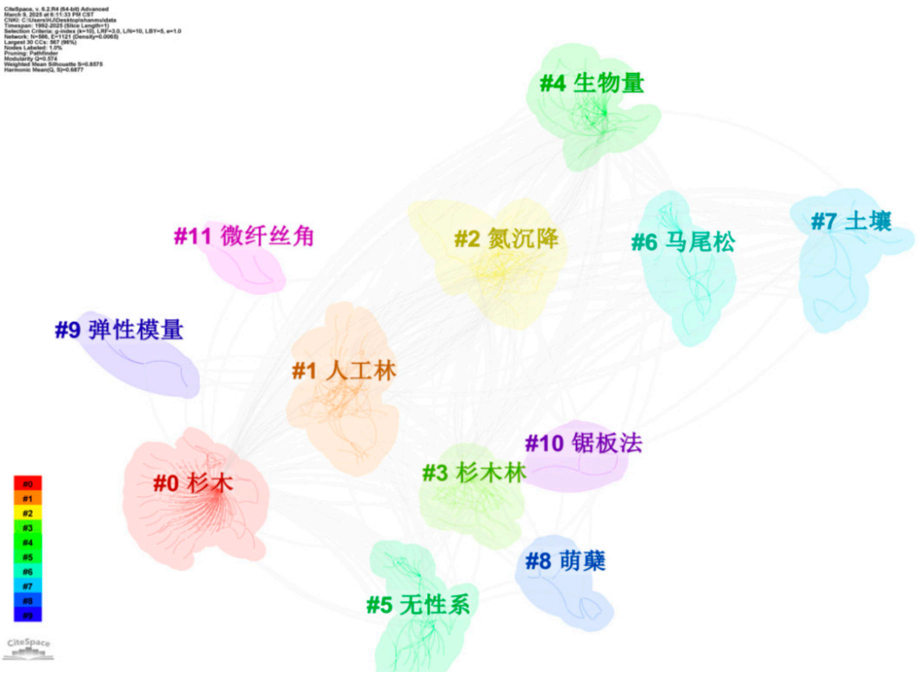

Figure S4 Keyword clustering of *C. lanceolata* in the three major Chinese databases. cluster #0, "*Cunninghamia lanceolata*", cluster #1, "plantation", cluster #2, "nitrogen deposition ", cluster #3, "C. lanceolata forest", cluster #4, "biomass", cluster #5, "clone", cluster #6, "*Pinus massoniana* Lamb.", cluster #7, "soil", cluster #8, "sprout tillers", cluster #9, "modulus of elasticity ", cluster #10, "sawmilling technique", cluster #11, "Microfibril Angle (MFA)".

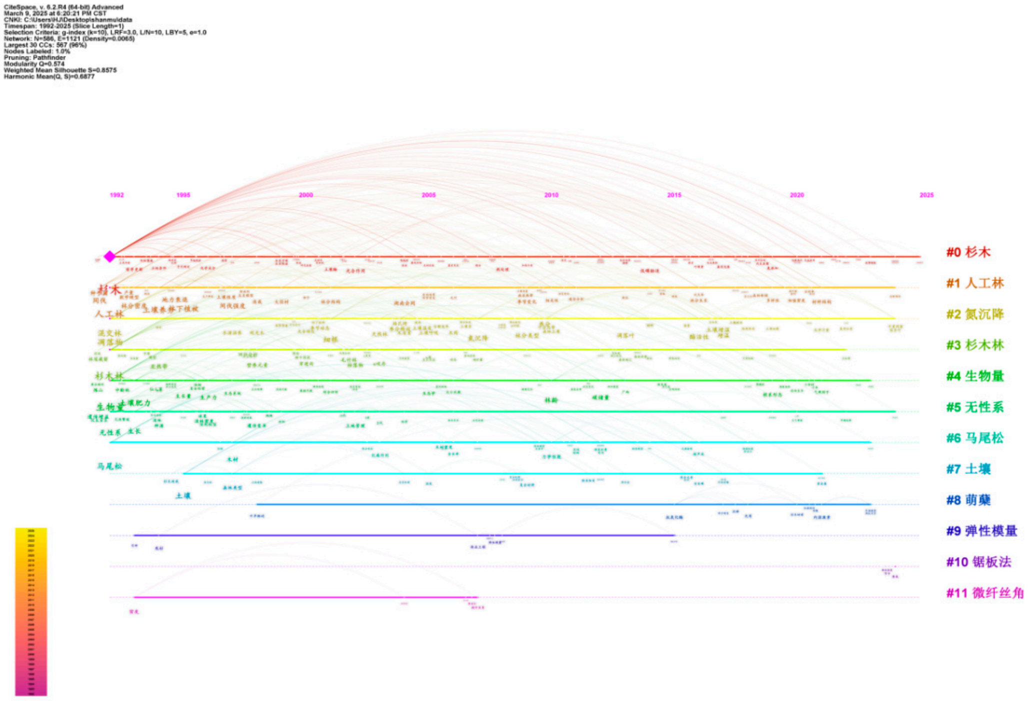

Figure S5 Keyword timing distribution of *C. lanceolata* in the three major Chinese database.
